# Florida Medical Association

**Published:** 1904-05

**Authors:** 


### SOCIETY REPORTS.

### FLORIDA MEDICAL ASSOCIATION.

Live Oak, Fla., April 22.

An amendment to the constitution and by-laws of the Florida Medical Association, making Jacksonville the permanent meeting place, has been submitted, and it has no opposition. Jacksonville also gets the president of the association for the ensuing year—Dr. E. N. Liell.

At yesterday's forenoon session the nominating committee of the house of delegates made the following report for officers for the ensuing year, which was adopted and said officers duly elected :

President—E. N. Liell, Jacksonville.

First Vice-President—J. W. Jackson, Miami.

Second Vice-President—J. F. McKinstry, Gainesville.

Third Vice-President—J. S. Holmes, Tampa.

Secretary and Treasurer—J. D. Fernandez, Jacksonville.

COUNCILLORS.

First District—J. Harris Pierpont, Pensacola.

Second District—H. E. Palmer, Tallahassee.

Third District—C. S. Brown, Live Oak.

Fourth District—J. H. Durkee, Jacksonville.

Fifth District—H. M. Taylor, Crystal River.

Sixth District—J. N. Fogarty, Key West.

Seventh District—W. L. Hughlette, Cocoa.

Eighth District—J. H. Hodges, Gainesville.

Resolutions were adopted strongly favoring a State Board of Medical Examiners.

Drs. W. L. Hughlette, J. H. Durkee and J. N. Fogarty were appointed as a committee on revision of the constitution and by-laws, to report at the next annual meeting.

A committee from the American Medical Association, relative to the importance of a uniform system of registration of vital statistics, was referred to the committee on legislation to prepare a bill to be presented at the next session of the Florida legislature and to urge its passage.

Following papers were read and discussed:

Surgery—Dr. J. N. Fogarty, chairman. Treatment of Trachoma, by Dr. J. H. Claiborne of New York. Report of a Case of Hepatic Abscess, and Report of a Case of Strangulated Hernia, by Dr. J. N. Fogarty of Key West. Remarks on the Pathology and Treatment of Appendix Abscesses, with Report of an Illustrative Case, by Dr. Carey P. Rogers, Jacksonville.

Medicine—Dr. Arthur L. Izlar, chairman. Paper on Anchylostomiasis, with Exhibition of the Parasites, by Dr. Aug. E. Unter, Greenville. Paper on Static Electricity, by Dr. Will White, Live Oak. Treatment of Affections of the Skin by the X-ray; High Frequency and Brush Discharges from a Static Machine, by Dr. R. P. Izlar, Waycross, Ga. Cerebro-Spinal Fever at Madison, with Report of Cases, by Dr. A. L. Blalock, Madison. A Few Observations on Typhoid Fever, by Dr. J. D. Love, Jacksonville.

Diseases of Children—Dr. J. D. Bennett, chairman.

Gynecology—Dr. L. M. Anderson, chairman. Ovarian Abscess; Sarcoma of Breast; Report of Rare Cases Operated Upon, with Specimens, by Dr. Edward M. Liell, Jacksonville. Leucorrhea in Married Women, by Dr. L. M. Anderson, Jasper. Subinvolution of the Uterus, by Dr. R. L. Goodbred, Mayo.

Hygiene—Dr. Eduardo Andrade, chairman. Some Remarks on the Bacillus of Diphtheria and Anisokoria (inequality of the pupils), by Dr. Eduardo Andrade, Jacksonville. Paper on Cerebro-Spinal Fever (?) at Madison. A Report on the Recent Epidemic, by Dr. Hiram Byrd, Madison.

Following resolution was adopted and the Association adjourned *sine die:*

"*Resolved*, That the Florida Medical Association hereby expresses sincere gratitude to the railroads of the State and the hotels of Live Oak for courtesies extended, to the citizens of Live Oak and the Suwannee County Medical Society for their free-handed, large-hearted hospitality, to the committee of arrangements, of which Dr. T. S. Anderson is chairman, for making the social features of the Association a success, and to Col. Frank Drew of the Suwannee and San Pedro Railroad for his special attention to the profession, for furnising his private car for excursion to Half Moon Lake."

The excursion to Half Moon Lake was greatly enjoyed and there the Association was splendidly entertained at an old-fashioned barbecue.

